# Return to Work After Surgery for Cervical Radiculopathy: Prospective Data From a Swedish Nationwide Cohort of 3929 Patients

**DOI:** 10.1227/neu.0000000000003927

**Published:** 2026-02-03

**Authors:** Victor Gabriel El-Hajj, Marcus Roland Victor Gustafsson, Mateo Tomás Fariña Núñez, Victor E. Staartjes, Erik Edström, Adrian Elmi-Terander

**Affiliations:** 1 Department of Clinical Neuroscience, Karolinska Institutet, Stockholm, Sweden;; 2 Department of Orthopaedic Surgery, Neurosurgery and Spine Surgery, Schulthess Clinic, Zurich, Switzerland;; 3 Machine Intelligence in Clinical Neuroscience and Microsurgical Neuroanatomy (MICN) Laboratory, Department of Neurosurgery, Clinical Neuroscience Center, University Hospital Zurich, University of Zurich, Zurich, Switzerland;; 4 Capio Spine Center Stockholm, Upplands Väsby, Sweden;; 5 Department of Medical Sciences, Örebro University, Örebro, Sweden;; 6 Department of Surgical Sciences, Uppsala University, Uppsala, Sweden

**Keywords:** Return to work, Spine surgery, Cervical radiculopathy

## Abstract

**BACKGROUND AND OBJECTIVES:**

Surgical decompression is commonly required to relieve symptoms of cervical radiculopathy and allow for functional recovery. There are only a handful of studies analyzing return-to-work (RTW) outcomes after cervical spine surgery for cervical radiculopathy. This study seeks to elucidate RTW outcomes and to identify predictors preventing RTW in patients surgically treated for cervical radiculopathy in a Swedish nationwide prospective registry.

**METHODS:**

A nationwide cohort analysis was conducted using prospectively gathered data from the Swedish Spine Registry. All patients with documented postoperative outcomes focusing on 1-year RTW rates were included. To identify predictive factors influencing RTW at 1 year postoperatively, separate univariable and multivariable logistic regression models were developed, incorporating demographic, functional and clinical, as well as preoperative and postoperative data, and occupational characteristics.

**RESULTS:**

A total of 3929 patients were included with an average age of 49.5 years, with most patients working in moderate- or high-intensity jobs and nearly half on sick leave before surgery. Most surgeries were elective, and an anterior approach was preferred. At the 1-year mark after surgery, 85% of patients had returned to work, with full-time RTW reaching 67% and part-time RTW 18%. In this cohort, 15% had not returned to work at all. Higher age, previous cervical spine surgery, high job intensity, preoperative full-time sick leave and full-time sickness benefits, longer preoperative arm pain durations as well as higher preoperative Neck Disability Index, and lower EuroQOL 5 dimensions scores were independently associated with a reduced likelihood of RTW at 1 year postoperatively.

**CONCLUSION:**

Eighty-five percent of the patients surgically treated for radiculopathy RTW within 1 year. Higher age, jobs with greater physical demands, longer duration of arm pain, higher preoperative neck disability index and lower EuroQOL 5 dimensions scores, as well as being on full-time sick leave, were all linked to a reduced chance of RTW.

## Graphical abstract

**Figure:**
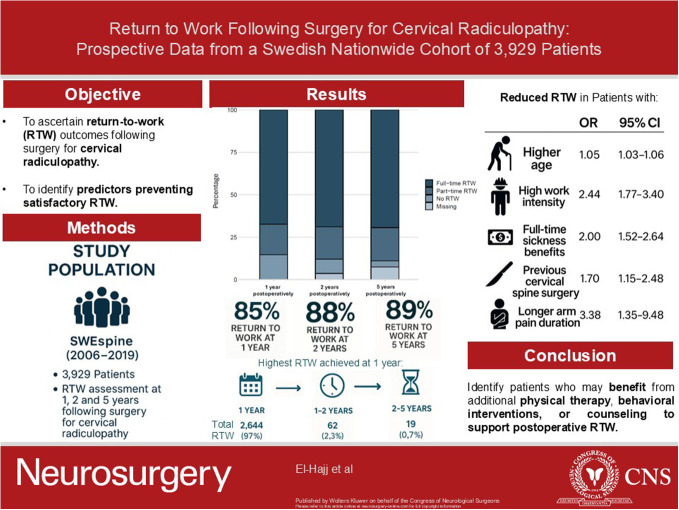

**ABBREVIATIONS:**
EQ-5D: EuroQOL 5 dimensions
NDI: Neck Disability Index
RTW: return to work
Swespine: Swedish spine registry.

Cervical radiculopathy, secondary to degenerative impingement of nerve roots, manifests as neck and neuropathic arm pain, often accompanied by sensorimotor symptoms and functional impairments reflecting the spinal level affected.^1,2^ Surgical decompression is commonly required to relieve symptoms and allow for functional recovery in individuals who experience persistent, severe symptoms that do not respond to conservative therapies.^2^ In general, cervical nerve root decompression is associated with good clinical outcomes and with a significant likelihood of relieving neurological impairments and neuropathic pain.^3-8^

Beyond the considerable personal suffering, radiculopathy imposes a substantial economic burden on society, largely attributable to lost productivity. Studies on the impact of chronic pain identify a considerable socioeconomic cost secondary to loss of ability to work.^9^ Consequently, return to work (RTW) is a relevant measure of surgical success, as it not only improves patients' quality of life but also reduces broader socioeconomic impacts.^10,11^ Despite significant postoperative improvements in pain and disability, a portion of patients still do not RTW.^12,13^ This has been shown in several studies studying RTW in patients undergoing lumbar spine surgery for acute or chronic back pain.^14-18^

However, only a handful of studies have assessed the predictive factors of reduced RTW after surgery for cervical radiculopathy.^12,19,20^ Using a nationwide prospective registry, this study aims to identify the predictors preventing RTW in patients surgically treated for cervical radiculopathy.

## METHODS

### Study Design

This analysis used data from the Swedish Spine Registry (Swespine),^21-27^ a prospective multicenter registry established to evaluate surgical outcomes for cervical spondylosis in Sweden. Swespine has a coverage of 98% (46 of 47 spinal clinics), the completeness is approximately 75% (30%-90%), and the 1-year follow up is performed in approximately 75%, covering all types of spinal surgical procedures. Swespine uses standardized protocols for enrollment and data collection. This study was approved by the Swedish National Ethics Review Authority (Dnr 2020-00193, and 2021-04773), which waived the requirement for informed consent in accordance with Swedish legislation.

### Patient Population and Variables

Inclusion criteria were adults of working age with a confirmed diagnosis of cervical radiculopathy, who underwent surgery and were registered in the Swespine registry from 2006 to 2019. Although Sweden does not have a fixed retirement age, 65 years was still seen as the standard retirement age in Sweden during the study period. The pension is largely based on life-time earnings and consequently there is an incentive to work beyond the reference age for retirement. To qualify for Swedish sickness-benefit, the individual must be insured in the Swedish social security system, have worked full time for at least 6 months, have a doctor assess and find a reduced ability to work, and finally the Swedish Social Insurance Agency must approve the benefit. The compensation is 80% of the preceding income up to about 60 000 USD per year and declines after 12 months. Patients older than 64 years were hence excluded from the analysis. Patients who were unemployed at the time of surgery were excluded from the analysis. Patients with incomplete records, precluding analysis of RTW at 1-year and its predictors, were also excluded. Patient-reported outcome measures included the Numeric Rating Scale for neck and arm pain severity, the EuroQOL 5 dimensions (EQ-5D) index, the EQ visual analogue scale, and the Neck Disability Index (NDI). Work intensity was defined as low (equivalent to office duties), moderate (a combination of administrative and physical duties, ie, working in a store), and high (heavy physical labor in industry, agriculture of transport sections). The main objective of this study was to evaluate the rate of RTW within 1 year after surgery and to determine the independent predictors preventing RTW. RTW was classified as either complete, partial, or no RTW, as reported by the patient.

### Statistical Analysis

Continuous variables were described using means and 95% CIs, whereas categorical variables were presented as frequencies and percentages. To identify factors associated with RTW at 12 months after surgery, separate univariable logistic regression models were constructed. These models accounted for a comprehensive range of preoperative, intraoperative, and occupational factors. To identify independent predictors, a multivariable logistic regression model for 12-month RTW was then established based on univariable significance (univariable *P* < .10) and excluding multicollinear variables. Statistical significance was determined with a *P* < .05. All statistical analyses were performed using the latest version of R Statistical software.

## RESULTS

### Preoperative Clinical Characteristics and Surgical Procedures

**FIGURE 1.**
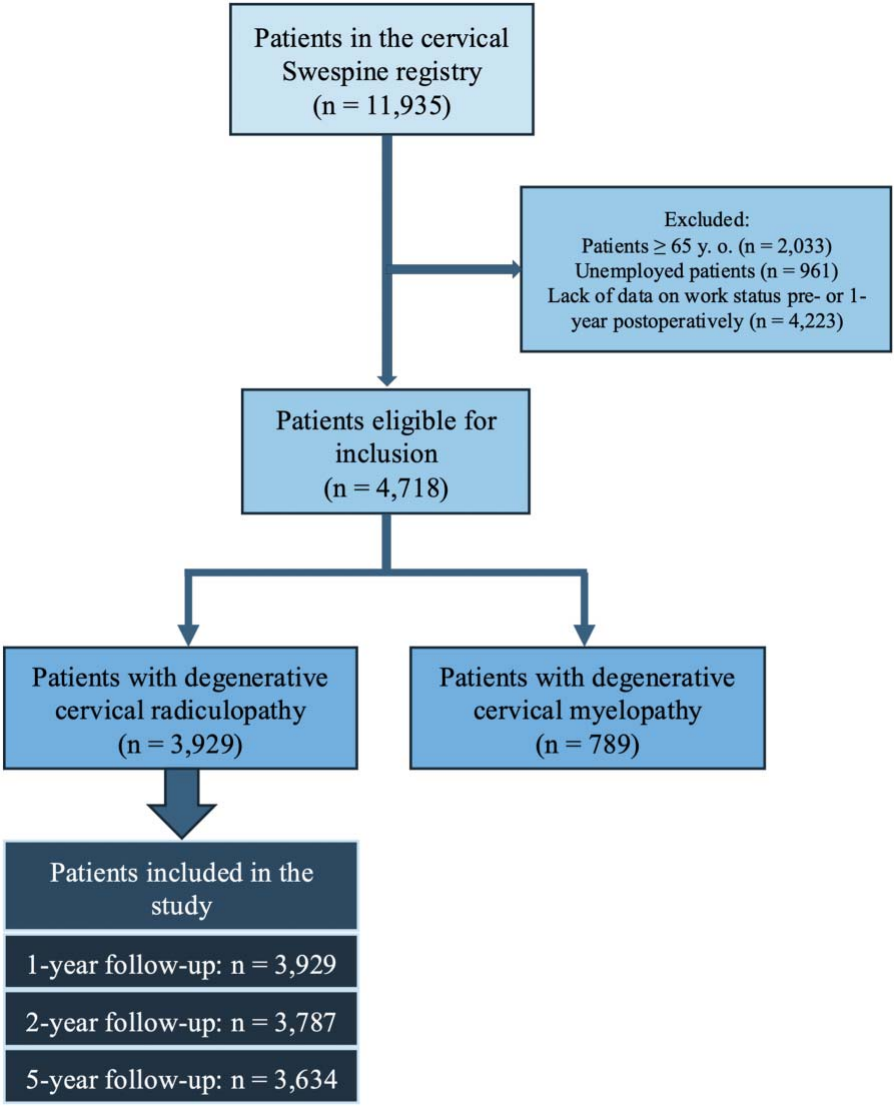
Flow chart illustrating the inclusion of patients in this study.

Among the 3929 individuals included in the study (Figure 1), the average age was 49.5 years, and 49% were male. Difficulties with fine motor skills were reported by 66% of patients, and neck or arm pain for more than 2 years by 42% and 31%, respectively. Before surgery, more than half of the cohort (52%) used pain medication daily and 15% used opioids. Limited mobility was noted by 18.5% of patients, who were unable to walk more than 1 kilometer. Most procedures (99%) were performed electively. The most frequently conducted operation was an anterior cervical diskectomy and fusion. Although a significant proportion of patients (49%) were on sick leave before surgery, only 17% were receiving full-time sickness benefits (Table 1).

**TABLE 1.** Baseline Demographic and Clinical Characteristics and Performed Surgical Procedures

No. of patients included	N = 3929
Age	49.53 (48.32-50.81)
BMI	26.82 (26.01-27.72)
Male sex, n (%)	1942 (49)
Smoker, n (%)	531 (14)
Previous cervical spine surgery, n (%)	403 (10)
Work intensity, n (%)	
Low	1182 (30)
Moderate	1553 (40)
High	1194 (30)
Preoperative sick leave, n (%)	
None	2006 (51)
Because of neck/arm issues (full-time)	1307 (33)
Because of neck/arm issues (part-time)	534 (14)
Because of other cause	82 (2.1)
Sick leave benefit, n (%)	
None	2793 (71)
Full-time sick leave benefit	686 (17)
Part-time sick leave benefit	450 (11)
Preoperative neck pain duration, n (%)	
<3 mo	113 (2.9)
3-12 mo	1105 (28)
1-2 y	875 (23)
>2 y	1623 (42)
No neck pain	164 (4.2)
Preoperative arm pain duration, n (%)	
<3 mo	169 (4.3)
3-12 mo	1387 (36)
1-2 y	995 (26)
>2 y	1218 (31)
No arm pain	118 (3.0)
Preoperative pain medication use, n (%)	
None	622 (16)
Regularly	2036 (52)
Occasionally	1271 (32)
Preoperative opioid medication use, n (%)	597 (15)
Length of walk at normal pace, n (%)	
<100 m	94 (2.4)
100-500 m	235 (6.1)
0.5-1 km	459 (12)
>1 km	3063 (80)
Preoperative fine motor issues, n (%)	2540 (66)
Type of procedure, n (%)	
Elective	3633 (99)
Nonelective	49 (1.3)
Approach, n (%)	
Anterior	3317 (84)
Posterior	612 (16)
Procedure, n (%)	
Diskectomy	3054 (78)
Foraminotomy	612 (16)
Arthroplasty	210 (5.3)
Corpectomy	53 (1.3)
Use of fixation	3306 (84)
Highest level operated, n (%)	
C2	3 (<0.1)
C3	7 (0.2)
C4	152 (4.0)
C5	487 (13)
C6	2129 (56)
C7	994 (26)
No. of operated levels per surgery	1.44 (1.02-1.98)

BMI, body mass index.

### Primary Outcome—RTW

**FIGURE 2.**
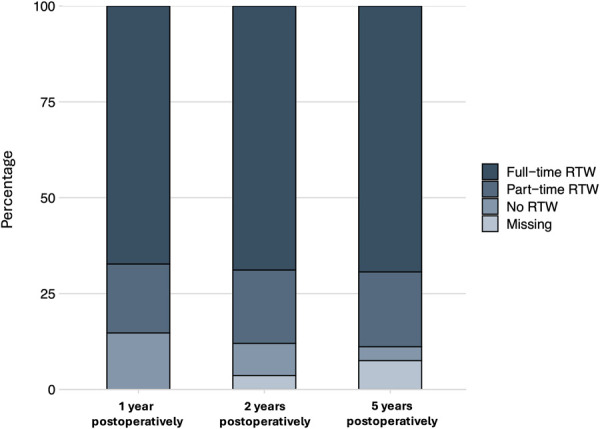
Bar charts showing the rates of RTW at 1, 2, and 5 years after surgery for cervical radiculopathy. RTW, return to work.

Of the 3929 patients studied, 56% required less than 3 months of fully paid sick leave after surgery, whereas 28% needed between 3 and 6 months, and 7.1% were on sick leave for more than a year. At the 1-year mark after surgery, 85% of patients had RTW, with full-time RTW reaching 67% and part-time RTW 18%. This figure increased slightly to 88% 2 years postoperatively, with the proportion of individuals who had not resumed work dropping to 8.4%. Five years after surgery, long-term results remained consistent, with 89% of patients having RTW and only 3.6% still not working (Figure 2). Notably, 97% of RTW occurred within the first year, whereas 3% required between 1 and 5 years (Table 2).

**TABLE 2.** RTW Analysis

No. of patients included	N = 3929
Postoperative length of fully paid sick leave, n (%)	
<3 mo	2196 (56)
3-6 mo	1092 (28)
6-9 mo	204 (5.2)
9-12 mo	137 (3.5)
>1 y	278 (7.1)
Work status at 1 y postoperatively, n (%)	
Full-time RTW	2644 (67)
Part-time RTW	706 (18)
No RTW	579 (15)
Work status at 2 y postoperatively, n (%)	
Full-time RTW	2706 (69)
Part-time RTW	751 (19)
No RTW	330 (8.4)
Missing	142 (3.6)
Work status at 5 y postoperatively, n (%)	
Full-time RTW	2725 (69)
Part-time RTW	767 (20)
No RTW	142 (3.6)
Missing	295 (7.5)

RTW, return to work.

### Factors Predicting Failure to RTW

Higher age is linked to a lower likelihood of RTW (odds ratio [OR] = 1.05; 95% CI: 1.03-1.06; *P* < .001). A history of previous cervical spine surgery reduces the likelihood of RTW in both univariable and multivariable analyses (OR = 1.70; 95% CI: 1.15-2.48; *P* = .004). High-intensity work significantly raises the risk of not returning to work (OR = 2.44; 95% CI: 1.77-3.40; *P* < .001), whereas moderate-intensity work presents a comparatively smaller risk (OR = 1.60; 95% CI: 1.17-2.21; *P* = .004). Patients who were on full-time sick leave before surgery because of neck or arm pain were less likely to RTW (OR = 2.10; 95% CI: 1.57-2.83; *P* < .001), as were those receiving full-time sick-leave benefits (OR = 2.00; 95% CI: 1.52-2.64; *P* < .001). Individuals with preoperative neck pain lasting more than 2 years had a markedly reduced chance of RTW (OR = 1.59; 95% CI: 0.52-4.83; *P* = .008); however, this was only significant in the univariate analysis. The multivariate analysis showed a similar association with prolonged arm pain and lower RTW rates (OR = 3.38; 95% CI: 1.35-9.48; *P* = .014). On multivariable analysis, decreased walking distance at normal pace was significantly associated with an increased likelihood of failed RTW. Compared with the reference group (>1 km), those able to walk 0.5-1 km had an OR of 1.77 (95% CI: 1.25-2.38; *P* < .001), and those able to walk 100-500 m had an OR of 2.84 (95% CI: 1.79-3.92; *P* < .001). Although individuals able to walk less than 100 m had an elevated odds for failure to RTW (OR: 1.78), this association did not reach statistical significance on multivariable regression (95% CI: 0.94-3.27; *P* = .069). A lower EQ-5D index score (OR = 0.65; 95% CI: 0.32-0.84; *P* = .007) and higher NDI score (OR = 1.02; 95% CI: 1.01-1.03; *P* < .001) were both strong predictors of failure to RTW (Table 3).

**TABLE 3.** Predictors of Failure to RTW With 1 year Follow-Up

Regression	Univariable	Multivariable		
Characteristic	*P* value	OR	95% CI	*P* value
Age	**<.001**	1.05	1.03-1.06	**<.001**
BMI	.072	0.99	0.96-1.02	.43
Male sex	**.030**	0.90	0.71-1.14	.38
Smoker	**<.001**	1.28	0.95-1.70	.10
Previous cervical spine surgery	**.004**	1.70	1.15-2.48	**.006**
Work intensity				
Low	REF	REF	REF	REF
Moderate	**<.001**	1.60	1.17-2.21	**.004**
High	**<.001**	2.44	1.77-3.40	**<.001**
Preoperative sick leave				
None	REF	REF	REF	REF
Because of neck/arm issues (full-time)	**<.001**	2.10	1.57-2.83	**<.001**
Because of neck/arm issues (part-time)	.14	1.04	0.67-1.57	.86
Because of other cause	**<.001**	4.29	2.27-7.98	**<.001**
Sick leave benefits				
None	REF	REF	REF	REF
Full-time sick leave benefits	**<.001**	2.00	1.52-2.64	**<.001**
Part-time sick leave benefits	.45	0.62	0.40-1.54	.89
Preoperative neck pain duration				
<3 mo	REF	REF	REF	REF
3-12 mo	.36	1.26	0.43-3.77	.67
1-2 y	**.048**	1.67	0.55-5.13	.36
>2 y	**.008**	1.59	0.54-4.83	.40
No neck pain	.26	1.89	0.57-6.25	.29
Preoperative arm pain duration				
<3 mo	REF	REF	REF	REF
3-12 mo	.16	1.85	0.76-5.10	.20
1-2 y	**.011**	2.07	0.82-5.84	.14
>2 y	**<.001**	3.38	1.35-9.48	**.014**
No arm pain	.62	2.96	0.91-9.99	.074
Preoperative pain medication				
None	REF	—	—	—
Regularly	.069	—	—	—
Occasionally	.19	—	—	—
Preoperative opioid use	>.99	—	—	—
Length of walk at normal pace				
>1 km	REF	REF	REF	REF
0.5-1 km	**<.001**	1.77	1.25-2.38	**<.001**
100-500 m	**<.001**	2.84	1.79-3.92	**<.001**
<100 m	**<.001**	1.78	0.94-3.27	.069
Preoperative fine motor deficits	**<.001**	1.17	0.89-1.54	.27
Type of procedure				
Elective	REF	—	—	—
Nonelective	.73	—	—	—
Surgical approach				
Anterior	REF	—	—	—
Posterior	.22	—	—	—
Surgical procedure				
Diskectomy	REF	REF	REF	REF
Foraminotomy	.22	0.87	0.63-1.18	.38
Corpectomy	**.006**	1.71	0.69-3.93	.23
Arthroplasty	.23	0.93	0.54-1.52	.77
Fixation surgery	.45	—	—	—
Highest operated level^a^	**<.001**	0.87	0.73-1.04	.12
No. of operated levels	**<.001**	1.08	0.90-1.30	.40
Preoperative NRS neck pain score	**<.001**	0.97	0.90-1.03	.30
Preoperative NRS arm pain score	**<.001**	1.03	0.97-1.09	.37
Preoperative EQ-5D index	**<.001**	0.65	0.32-0.84	**.007**
Preoperative EQ-VAS score	**<.001**	—	—	—
Preoperative NDI	**<.001**	1.02	1.01-1.03	**<.001**

BMI, body mass index; EQ-5D, EuroQOL 5 dimensions; NDI, Neck Disability Index; NRS, Numeric Rating Scale; OR, odds ratio; REF, reference level; RTW, return to work; VAS, visual analogue scale.

a The highest operated vertebral level was encoded as a continuous variable by assigning each cervical vertebra (C2 to C7) a numerical value from 1 to 6, respectively.

Bold indicates *P* < .05.

### Impact of RTW on Patient-Reported Outcome Measures

At 1 year postoperatively, patients who RTW demonstrated significantly greater clinical improvement than those who did not (all *P* < .001, Table 4). Complete or partial resolution of neck and arm pain was more frequent among RTW patients (84% and 87%) compared with non-RTW patients (62% and 58%), who also had higher rates of persistent or worsened pain. Regular pain medication and opioid use were markedly lower in the RTW group (16% and 7.9%) than in those who did not RTW (50% and 17%). RTW patients also showed greater functional recovery, with larger mean improvements in NDI (−19.8 vs −7.7), EQ-5D (0.30 vs 0.12), and EQ-visual analogue scale (22.0 vs 6.3), and reported higher overall satisfaction (81% vs 48%).

**TABLE 4.** Table Demonstrating the Impact of RTW on Patient-Reported Outcomes

Variable	RTW, N = 3350	No RTW, N = 579	*P* value
Postoperative neck pain status at 1-y, n (%)			**<.001**
No preoperative neck pain	286 (8.6)	39 (6.8)	
Complete resolution	658 (20)	44 (7.7)	
Partial resolution	2114 (64)	308 (54)	
Unchanged	169 (5.1)	92 (16)	
Worse	100 (3.0)	88 (15)	
Postoperative arm pain status at 1-y, n (%)			**<.001**
No preoperative neck pain	116 (3.5)	15 (2.6)	
Complete resolution	1054 (32)	58 (10)	
Partial resolution	1816 (55)	277 (48)	
Unchanged	217 (6.5)	131 (23)	
Worse	118 (3.6)	94 (16)	
Pain medication use at 1-y postoperatively, n (%)			**<.001**
None	1615 (48)	120 (21)	
Regularly	548 (16)	288 (50)	
Occasionally	1187 (35)	171 (30)	
Opioid use at 1 y postoperatively	265 (7.9)	101 (17)	**<.001**
Postoperative fine motor status at 1 y, n (%)			**<.001**
No preoperative fine motor skill issues	793 (24)	63 (11)	
Complete resolution	666 (20)	30 (5.2)	
Partial resolution	1314 (40)	238 (41)	
Unchanged	403 (12)	159 (28)	
Worse	121 (3.7)	86 (15)	
Postoperative subjective satisfaction, n (%)			**<.001**
Satisfied	2672 (81)	274 (48)	
Undecided	461 (14)	191 (33)	
Not satisfied	157 (4.8)	106 (19)	
One-year post vs preoperative difference in			
NRS neck pain scores	−3.20 (2.91)	−1.74 (2.96)	**<.001**
NRS arm pain scores	−3.47 (3.10)	−1.87 (3.08)	**<.001**
EQ-5D index	0.30 (0.34)	0.12 (0.37)	**<.001**
EQ-VAS score	21.99 (25.25)	6.34 (25.61)	**<.001**
NDI	−19.83 (16.73)	−7.73 (18.19)	**<.001**

EQ-5D, EuroQOL 5 dimensions; NDI, Neck Disability Index; NRS, Numeric Rating Scale; RTW, return to work; VAS, visual analogue scale.

Significant *P* values are highlighted in bold character.

## DISCUSSION

Surgical treatment for cervical radiculopathy is intended to relieve symptoms and promote functional recovery. An important goal and measure of overall recovery is RTW. By analyzing outcomes in 3929 Swedish patients who underwent spine surgery for cervical radiculopathy, this study provides valuable long-term data on predictors preventing RTW. Preoperatively, 51% of patients were on sick leave, reflecting the significant impact of radiculopathy on employment and daily life. Postoperatively, 85% of patients resumed work within the first year, with 67% returning to full-time employment. By the second year, the RTW rate increased to 88%, and stabilized at 89% after 5 years. Among those who returned to full-time work, 97% did so within the first year, reflecting a typically swift recovery after spine surgery for cervical radiculopathy. The published literature regarding RTW rates after surgery for cervical radiculopathy is sparse, with more studies analyzing RTW after lumbar spine surgery, either for low back pain or lumbar radiculopathy.^14,15,28^ To our knowledge, this study is one of the largest studies worldwide focusing on postoperative RTW rates after surgery for cervical radiculopathy.

This study revealed that patients on full-time sick leave and receiving full-time sick-leave benefits preoperatively were significantly less likely to RTW, being twice as likely not to return compared with those without such benefits (OR = 2.00, *P* < .001). These observations align with the findings of Huysmans et al^14^ and Hara et al.^19^ Both studies, also conducted in a European welfare context, highlight the potential influence of social benefit systems on RTW. The Norwegian study of Hara et al^19^ analyzed RTW outcomes after surgery for cervical radiculopathy and found that about half of the patients resumed work within 4 months, and to 67% by 1 year. Surprisingly, the authors also found that RTW rates within the first year after surgery were even more strongly determined by the number of sick days before surgery than by the degree of postoperative clinical improvement.^19^ In the systematic review by Huysmans et al,^14^ the authors found that the most important predictor of RTW 2 years later was having 90 or fewer sick days in the year before surgery, regardless of preoperative disability. The authors concluded that the number of sick days should be carefully considered when evaluating surgical candidates, especially when RTW is an important treatment goal. Other factors negatively influencing RTW included higher age, female sex, low educational level, and receipt of long-term medical benefits, suggesting that socioeconomic factors significantly impact RTW. Studying elective cervical spine surgery in the United States, Chanbour et al^29^ also identified more physically demanding work and receiving full-time sickness benefits or disability insurance before surgery to be associated with a markedly lower likelihood of RTW.

Several other factors influenced RTW rates after surgery for cervical radiculopathy, in this study. Older patients were less likely to RTW. Extended durations of arm pain (over 2 years), higher NDI and lower EQ-5D scores, as well as previous cervical spine procedures, were also linked to lower RTW rates. Persistent postoperative neck pain was associated with poorer RTW outcomes in another study.^30^ Consistent with findings reported by Singh et al,^28^ occupational factors were especially important, as patients in physically demanding jobs were more than twice as likely to not RTW compared with those in lighter jobs. Overall, the predictive factors for not RTW are consistent with recent studies, which have repeatedly identified age, job intensity, and preoperative work status as determinants of RTW after cervical spine surgery.^13,31-33^

On another note, patients who RTW showed greater pain relief, functional improvement, and satisfaction than those who did not (*P* < .001), indicating that successful RTW closely parallels overall postoperative recovery and health-related quality of life. Similar observations have been shared by previous authors. For instance, in the study by Hara et al,^19^ the authors found that larger proportions of patients who returned to work achieved minimal clinically important changes in function and pain. In addition, in their study,^19^ patients who had returned to work were also more likely to have recovered completely or to a large extent, as compared with those who did not (78.0% vs 45.2%; *P* < .001).

Finally, it is worth noting that the Swedish system provides generous and standardized sick-leave compensation, which may facilitate extended work absences compared with countries such as the United States, where employer-based or private insurance systems predominate and economic pressures may prompt earlier RTW. By contrast, some Asian countries, including Japan and South Korea, offer more limited short-term benefits and stronger workplace reintegration programs. These differences highlight that RTW behavior is partly shaped by the surrounding socioeconomic and policy environment,^34,35^ which may limit direct extrapolation of our findings to other health care systems.

### Limitations

This study is based on prospectively collected registry data from the Swespine. Consequently, this study is limited to the data contained therein. Although the loss of data because of missingness may lead to significant selection bias, hampering the internal validity of the findings, previous study conducted on the Swespine registry have determined that loss to follow-up did not bias the observations.^36^ In addition, although the national coverage of 98% of the Swespine may be one of the largest in the world, this data may not accurately represent other populations outside Sweden. Especially because the RTW analysis performed in this study is closely tied to the Swedish Social Insurance Agency, which in its function may differ drastically from similar agencies in other countries. As such, the external validity of these results in other populations and pertaining to different retirement systems need further investigation. Finally, variables that are not accounted by the Swespine registry may have acted as confounders, interfering with the results of the analysis. In particular, information regarding medical comorbidities, which may influence RTW, is not consistently available in the registry and therefore could not be included in this analysis.

## CONCLUSION

Most patients undergoing surgery for cervical radiculopathy RTW within the first year after the surgery. Higher age, previous cervical spine surgery, jobs with greater physical demands, longer duration of arm pain, higher preoperative NDI, lower EQ-5D index, full-time sick leave, and receiving full sick-leave benefits, appeared to be factors preventing RTW. Recognizing these risk factors can help guide patient selection and identify those who may benefit from additional physical therapy, behavioral interventions, or counseling to support their RTW.
